# Association of Serum IL-17A and IL-23 Levels With Alopecia Areata: A Cross-Sectional Comparative Study

**DOI:** 10.7759/cureus.100011

**Published:** 2025-12-24

**Authors:** Md. Shihab Talukder, A.T.M. Asaduzzaman, Mohammad Abul Kalam Azad, Mohammad Jamal Uddin, Pranam Swapan Dash, Wasi Deen Ahmed

**Affiliations:** 1 Dermatology, Directorate General of Health Services (DGHS), Dhaka, BGD; 2 Dermatology, Bangladesh Medical University, Dhaka, BGD; 3 Rheumatology, Bangladesh Medical University, Dhaka, BGD

**Keywords:** alopecia areata, autoimmune disease, cytokine assay, il-17a, il-23

## Abstract

Background and objective: Alopecia areata (AA) is an autoimmune, non-scarring hair loss disorder in which T helper 17 (Th17) cells play a key role. Interleukin-23 (IL-23) promotes the differentiation and expansion of Th17 cells from naïve CD4+ T cells. This study aimed to evaluate the association between serum IL-17A and IL-23 levels and disease severity in patients with AA.

Methods: A cross-sectional comparative study was conducted at the Department of Dermatology and Venereology, Bangladesh Medical University, Dhaka, from October 2022 to September 2024. Forty-three AA patients and 43 age- and sex-matched healthy controls were enrolled. Serum IL-17A and IL-23 levels were measured using enzyme-linked immunosorbent assay (ELISA) (Elabscience, USA). Disease severity was assessed using the Severity of Alopecia Tool (SALT) score. Data were analyzed with IBM SPSS Statistics for Windows, Version 26 (released 2018; IBM Corp., Armonk, New York, United States) using chi-square, t-test, Mann-Whitney U, and Spearman’s correlation tests, with p<0.05 considered statistically significant.

Results: Serum IL-17A and IL-23 levels were significantly higher in AA patients than controls (IL-17A: 39.4 ± 38.3 vs. 13.7 ± 10.7 pg/mL; IL-23: 48.2 ± 57.0 vs. 15.5 ± 15.5 pg/mL; both p<0.001). IL-17A correlated positively with disease severity (p=0.016), increasing from 21.17 ± 33.26 pg/mL in mild cases (S1, <25% hair loss) to 63.47 ± 50.33 pg/mL in severe cases (S4, 75-99% hair loss). IL-17A levels varied across clinical subtypes (single patch: 20.70 ± 24.46 pg/mL; sisaipho: 53.37 ± 1.33 pg/mL; p=0.044). IL-23 levels remained elevated regardless of disease severity (p=0.118) or clinical subtype (p=0.378). A significant positive correlation existed between IL-17A and IL-23 (p<0.001).

Conclusions: Elevated IL-17A levels are associated with AA severity and differ across clinical subtypes, suggesting a role in disease progression and phenotype variation. IL-23 is consistently elevated in AA, highlighting its potential as a biomarker for disease activity. These findings provide insight into cytokine dysregulation in AA and may inform future therapeutic strategies.

## Introduction

Alopecia areata (AA) is an organ-specific autoimmune disorder that primarily affects hair follicles, presenting as sudden, well-demarcated, round or oval patches of non-scarring hair loss. The disease course is often unpredictable, with episodes of spontaneous remission and relapse [1]. The pathogenesis of AA is multifactorial, involving complex interactions among immunological, genetic, and environmental factors [2]. Although the precise etiology remains unclear, T cell-mediated autoimmunity targeting an unidentified hair follicle antigen is considered central to disease development. Active AA is characterized by perifollicular infiltration of CD4+ T cells and intrafollicular infiltration of CD8+ T cells, which mediate hair follicle damage [1]. Specialized CD4+ T cell subsets, including T helper (Th)1, Th2, Th17 cells, and regulatory T cells (Tregs), play crucial roles in autoimmune processes. Th17 cells, a distinct subset of T helper cells, secrete interleukin-17A (IL-17A) [3], a pro-inflammatory cytokine implicated in autoimmune tissue damage. Th1 and Th17 cytokines are hypothesized to promote immune-mediated attacks on hair follicles, causing premature cessation of the hair growth cycle and perpetuating the alopecia phenotype [4].

Interleukin-23 (IL-23), a member of the IL-12 cytokine family, promotes Th17 cell differentiation and pathogenicity. IL-23 signals via Janus kinase 2 (Jak2) and tyrosine kinase 2 (Tyk2) to activate signal transducer and activator of transcription 3 (STAT3) and STAT4 [5]. STAT3 regulates the transcription factor retinoic acid receptor-related orphan receptor γt (RORγt), which is essential for Th17 cell development and function [6]. Overexpression of RORγt enhances inflammatory cytokine and chemokine production by Th17 cells [7]. Consequently, IL-23 drives the expansion of pathogenic Th17 cells that secrete IL-17, IL-22, and TNF-α [8].

Elevated IL-17 levels have been implicated in multiple autoimmune and chronic inflammatory diseases, including systemic lupus erythematosus, rheumatoid arthritis, psoriasis, inflammatory bowel disease, multiple sclerosis, systemic sclerosis, and transplant rejection [1]. IL-17 activates STAT3, recruits leukocyte subsets, and induces the expression of pro-inflammatory cytokines (IL-6, IL-8, TNF-α), chemokines (CXCL1, CXCL2, CXCL3, CXCL5, CXCL8), metalloproteinases, and other inflammatory mediators [8,9]. Additionally, IL-17 upregulates ICAM-1 on fibroblasts and promotes dendritic cell maturation [8]. The pivotal role of Th17 cells and IL-23/IL-17A signaling in autoimmune inflammation: investigating their levels in AA patients may enhance understanding of disease pathophysiology and identify potential biomarkers for disease activity. This study aimed to assess serum IL-17A and IL-23 levels in patients with AA and evaluate their association with disease severity and clinical subtypes.

## Materials and methods

Study design and setting

This cross-sectional comparative study was conducted at the Department of Dermatology and Venereology, Bangladesh Medical University (BMU), Dhaka, Bangladesh, from October 2022 to September 2024. IRB approval was provided by Bangabandhu Sheikh Mujib Medical University or Bangladesh Medical University (BSMMU/2023/9889).

Study population

Participants were divided into two groups: Group A (patients) were clinically diagnosed cases of AA attending the outpatient department. Group B (controls) were apparently healthy individuals, including attendants, doctors, nurses, and other BMU staff.

Sample size and sampling technique

The sample size was calculated using formulas described by Chow et al., Julious, and Machin et al. [10-12]. A total of 43 participants per group (N=86) were included. Consecutive sampling was applied to recruit participants.

Inclusion and exclusion criteria

Group A had patients diagnosed with AA, all ages and sexes. Group B had healthy individuals of all ages and sexes. Exclusion criteria include patients who have received topical or systemic AA treatment or immunosuppressive therapy within the past three months. Pregnant or lactating women and individuals with known autoimmune, infectious, or inflammatory diseases, thyroid disorders, vitamin D deficiency, atopy, psoriasis, and obesity were excluded.

Ethical considerations

**Figure 1:**
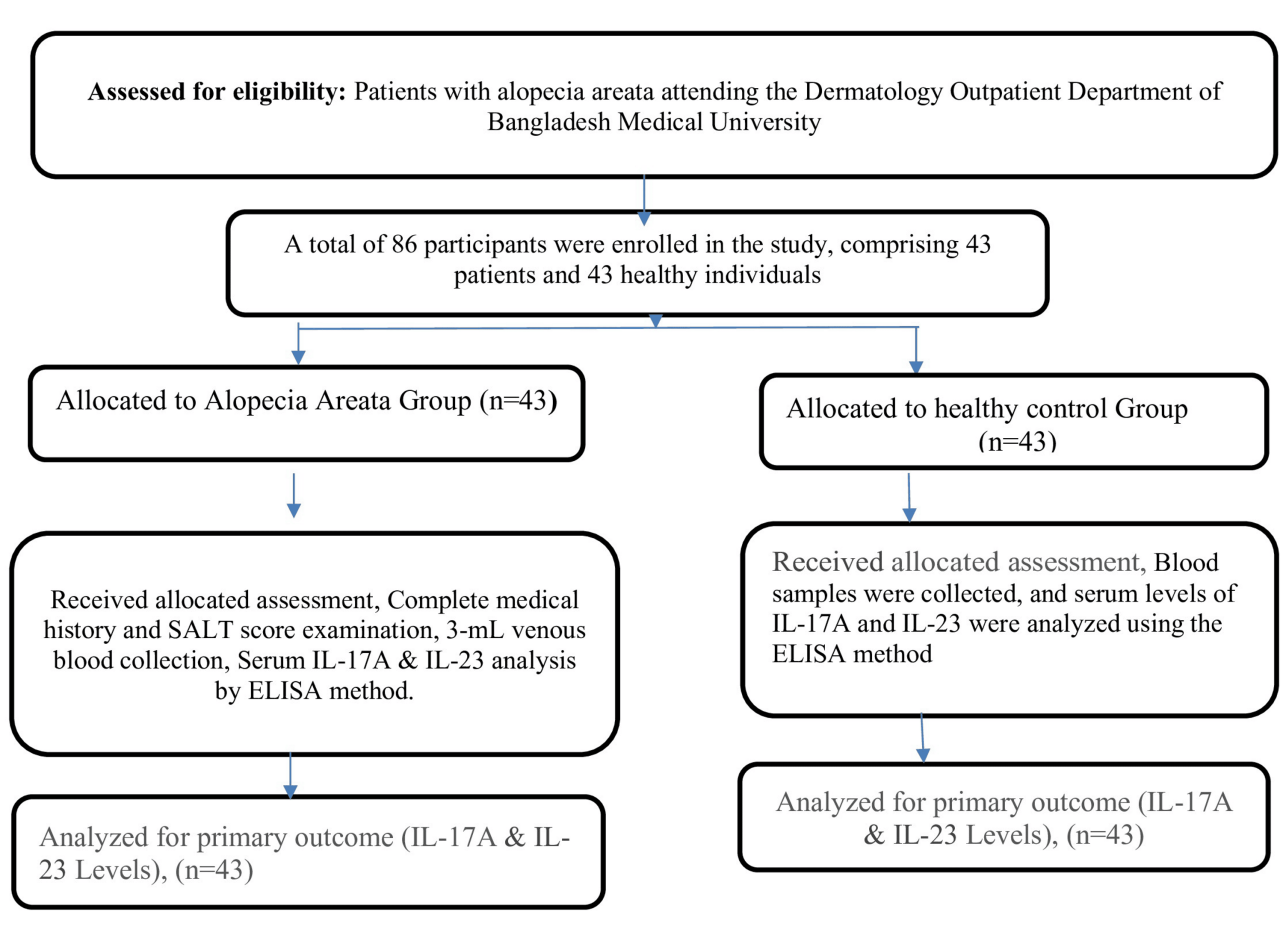
Flowsheet of study population SALT: Severity of Alopecia Tool; IL-17A: interleukin 17A; IL-23: interleukin 23; ELISA: enzyme-linked immunosorbent assay

The study was approved by the Institutional Review Board of BMU (IRB No: BSMMU/2023/9889). Written informed consent was obtained from all participants prior to enrollment. The flowsheet of the study population is given in Figure 1.

Clinical type and severity assessment

Diagnosis of AA was based on clinical history, physical examination, and trichoscopy. The severity of scalp hair loss was quantified using the Severity of Alopecia Tool (SALT) score [13]. Patients were further subclassified according to SALT categories (S0-S5) and body hair loss grades (B0-B2). The severity of hair loss was assessed by the SALT score. Subgrouping of patients into SALT subclasses was done as follows: scalp (S): S0, no hair loss; S1, <25% hair loss; S2, 25-49% hair loss; S3, 50-74% hair loss; S4, 75-99% hair loss, and S5, 100% hair loss [14]. Forty-three age- and gender-matched consenting healthy individuals were enrolled as controls.

Measurement of IL-17A and IL-23

Venous blood (3 mL) was collected from each participant, centrifuged at 4000 rpm for five minutes, and serum was stored at -20°C until analysis. Serum IL-17A and IL-23 levels were measured using commercial enzyme-linked immunosorbent assay (ELISA) kits (Elabscience, USA; IL-17A: E-EL-H0105, IL-23: E-EL-H0107) according to the manufacturer’s instructions. Optical density was read at 450 nm [15,16]. Measurement of cytokine levels of all study subjects was done on the same day with the same reagent.

Statistical analysis

After the collection of all the required data, the data were checked, verified for consistency, and tabulated using IBM SPSS Statistics for Windows, Version 26 (released 2018; IBM Corp., Armonk, New York, United States). Frequency and percentage were used to express categorical data. Mean and standard deviation were used to express continuous data. To determine the association between categorical data, a chi-square test was done. To determine the difference between the continuous data of two groups, an unpaired Student’s t-test was done. Spearman’s rank order coefficient analysis was done to see the correlation of serum IL-17A and IL-23 levels with age, age of onset, and duration of the disease, and the correlation of serum IL-17A with IL-23. The Mann-Whitney U test was performed to compare the two groups. The Kruskal-Wallis test was used for comparing among subgroups. For all statistical tests, a p-value <0.05 was considered statistically significant.

## Results

A total of 86 participants were included in the study, comprising 43 patients with AA (Group A) and 43 age- and sex-matched healthy controls (Group B).

Demographic characteristics

The majority of participants were aged 11-20 years (Group A: 13 (30.2%); Group B: 17 (39.5%)), followed by those aged 31-40 years (10 (23.3%) in each group) and 21-30 years (Group A: 9 (20.9%); Group B: 7 (16.3%)). The mean age was 24.5 ± 12.1 years in Group A and 23.4 ± 12.2 years in Group B, with no statistically significant difference between groups (p=0.670).

Regarding gender, Group A included 22 males (51.2%) and 21 females (48.8%), while Group B comprised 20 males (46.5%) and 23 females (53.5%), with no significant difference observed (p=0.666). These findings indicate that the two groups were comparable in terms of age and gender (Table 1).

**Table 1:** Demographic profile of two groups and total sample (n=86) Data were expressed as frequency, percentage, and mean ± SD. The p-value obtained by the unpaired Student’s t-test (a) and chi-square test (b), p<0.05, was considered as a level of significance. Group A: diagnosed patients with alopecia areata; Group B: apparently healthy individuals without alopecia areata.

Variables	Group A (Patients) (n=43) No. (%)	Group B (Healthy) (n=43) No. (%)	t/χ value	p-value
Age group (years)	-	-	-	-
<10	5 (11.6%)	4 (9.3%)	-	-
11-20	13 (30.2%)	17 (39.5%)	-	-
21-30	9 (20.9%)	7 (16.3%)	-	-
31-40	10 (23.3%)	10 (23.3%)	-	-
41-50	6 (14.0%)	5 (11.6%)	-	-
Mean ± SD Range	24.5 ± 12.1 (5-45) years	23.4 ± 12.2 (5-45) years	0.427	0.670^a^
Gender	-	-	-	-
Male, Female	22 (51.2%), 21 (48.8%)	20 (46.5%), 23 (53.5%)	0.186	0.666^b^
Total	43 (100.0%)	43 (100.0%)	-	-

Clinical characteristics of patients with AA

The duration of AA ranged from one to 144 months, with a mean duration of 24.8 ± 36.2 months (Table 2). The onset of disease occurred before the age of 16 years in 13 patients (30.2%), while 30 patients (69.8%) experienced onset after 16 years. The mean age of onset was 22.5 ± 11.9 years, with a median of 21.6 years (Table 2).

**Table 2:** Distribution of the study population by duration of disease, age of onset in group A (n=43) SD: standard deviation; M: months; Y: years; n: number; %: percentage

Variables	Category	n (%)	Mean ± SD	Median	Range
Disease duration (months)	<6 M	18 (41.9%)	-	-	-
	≥6 M	25 (58.1%)	-	-	-
-	Total	43 (100%)	24.8 ± 36.2	9.5	1–144
Age of onset (years)	<16 Y	13 (30.2%)	-	-	-
	≥16 Y	30 (69.8%)	-	-	-
-	Total	43 (100%)	22.5 ± 11.9	21.6	4–45

Based on clinical type, 11 patients (25.6%) presented with a single patch of AA, 21 (48.8%) with multiple patches, four (9.3%) with ophiasis, two (4.7%) with sisaipho, two (4.7%) with alopecia totalis (AT), and three (6.9%) with alopecia universalis (AU).

Regarding severity, 11 patients (25.5%) were classified as S1 (<25% scalp hair loss), 13 (30.2%) as S2 (25-49%), 10 (23.3%) as S3 (50-74%), seven (16.3%) as S4 (75-99%), and two (4.7%) as S5 (100%; AT/AU) (Table 3).

**Table 3:** Distribution of patients according to clinical type and severity of alopecia areata S: scalp hair loss; n: number; %: percentage; AA: alopecia areata. The “S” grading system (S1–S5) is used to classify the severity of scalp hair loss, based on the percentage of hair loss area.

Clinical Type	n (%)	Severity Grade	n (%)
Single patch AA	11 (25.6%)	S1	11 (25.5%)
Multiple AA	21 (48.8%)	S2	13 (30.2%)
Ophiasis	4 (9.3%)	S3	10 (23.3%)
Sisaipho	2 (4.7%)	S4	7 (16.3%)
Alopecia totalis (AT)	2 (4.7%)	S5	2 (4.7%)
Alopecia universalis AU)	3 (6.9%)	-	-
Total	43 (100%)	Total	43 (100%)

Serum levels of cytokines IL‐17A and IL‐23 were significantly raised (p < 0.001) in AA patients as compared to age‐ and sex‐matched healthy individuals (Table 4).

**Table 4:** Comparison of serum IL-17A and serum IL-23 level between two groups (n=43) Data were expressed as mean ± SD, median, and range. The Mann-Whitney U test was performed to compare the two groups. * Significant, min-max: minimum-maximum, ** = t-test. IL-17A: interleukin 17A; IL-23: interleukin 23; n: number; *Significant, Group A: diagnosed patients with alopecia areata; Group B: apparently healthy individuals.

Lab findings	Category	Group A (Case) n=43	Group B (Healthy) n=43	Z-value	p-value
Serum IL-17A	Mean ± SD	39.4 ± 38.3	13.7 ± 10.7	-3.884	<0.001*
	Median	19.6	10.7		-
	Range (min-max)	2.72-128	0.66-54.9		-
Serum IL-23	Mean ± SD	48.2 ± 57.0	15.5 ± 15.5	-2.9422	<0.001*
	Median	16.1	8.82		-
	Range (min-max)	1.25-181.4	0.93-75.5		-

**Figure 2:**
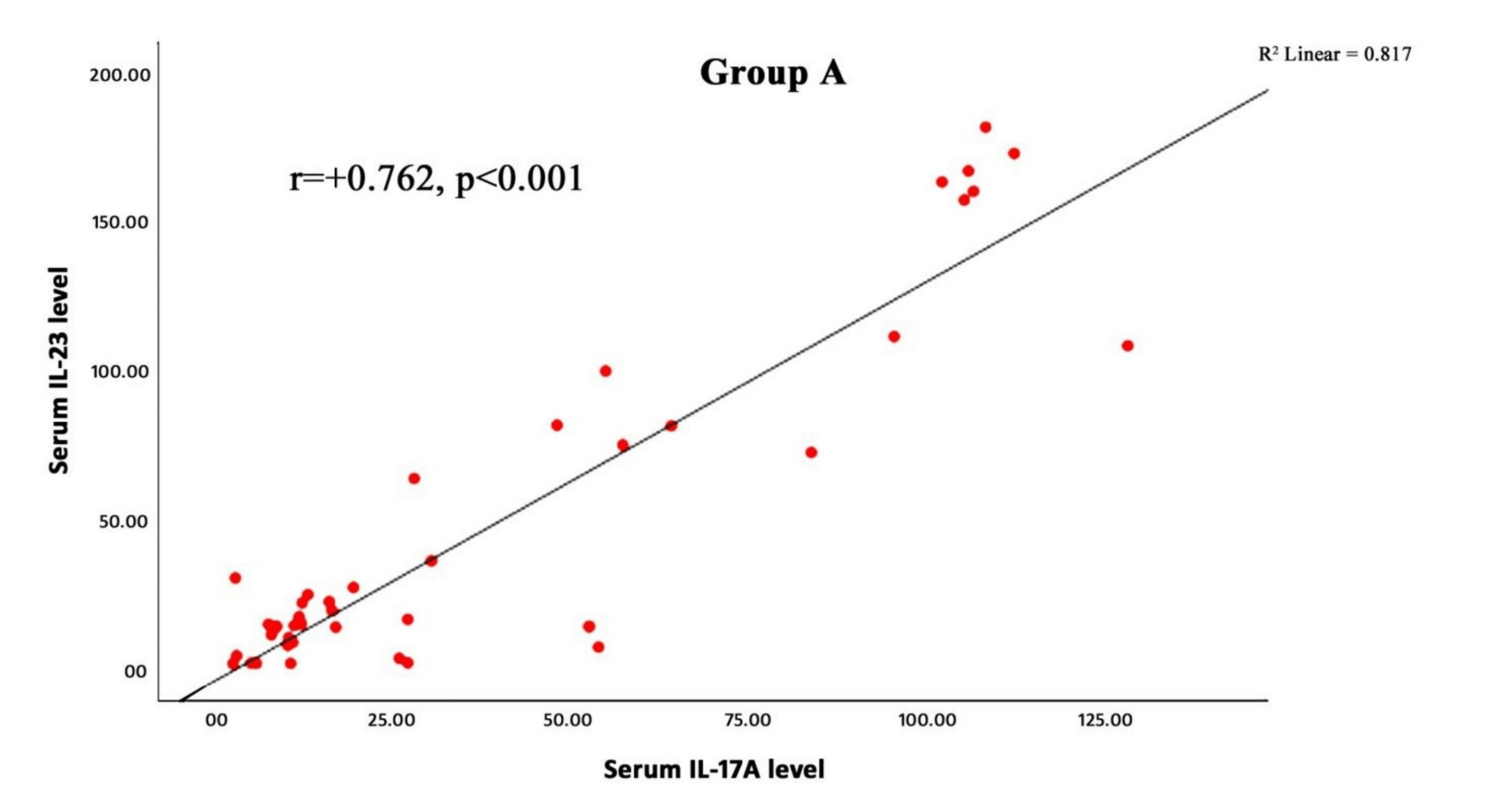
Scatter plot showing the correlation between serum IL-17A and IL-23 levels in patients with alopecia areata The X-axis shows serum IL-17A, and the Y-axis shows serum IL-23 concentration. The solid line represents the linear regression fit (r=+0.762, p < 0.001). A positive correlation was observed between serum IL-17A and IL-23 levels (Spearman’s ρ=0.762, p < 0.001).

A strong positive correlation was observed between serum IL-17A and IL-23 concentrations in patients with AA (Spearman’s ρ=0.762, r=+0.762, p < 0.001) (Figure 2). This finding indicates that higher serum IL-17A levels were significantly associated with higher IL-23 concentrations within the study population, suggesting a potential interrelated role of these cytokines in the immunopathogenesis of AA.

The serum levels of IL-17A showed a significant increase with the severity of AA (p=0.016, H=12.14). The mean IL-17A concentration rose progressively from 21.17 ± 33.26 pg/mL in S1, 35.36 ± 33.45 pg/mL in S2, and 52.04 ± 34.39 pg/mL in S3 to 63.47 ± 50.33 pg/mL in S4, followed by a decrease to 5.21 ± 3.51 pg/mL in S5, possibly due to the small sample size (n=2). Similarly, the median values increased from 10.5 pg/mL in S1 to 95.3 pg/mL in S4 before dropping to 5.21 pg/mL in S5. In contrast, serum IL-23 levels showed a non-significant upward trend with disease severity (p=0.118, H=7.36), with mean concentrations of 28.76 ± 58.04 pg/mL in S1, 31.87 ± 30.14 pg/mL in S2, 68.42 ± 60.92 pg/mL in S3, 90.84 ± 77.38 pg/mL in S4, and 7.83 ± 9.30 pg/mL in S5. The corresponding median IL-23 values were 9.9, 19.0, 69.1, 111.1, and 7.8 pg/mL for S1 to S5, respectively. Overall, IL-17A levels were significantly associated with AA severity, while IL-23 levels demonstrated an increasing but statistically non-significant trend, as shown in Table 5.

**Table 5:** Comparison of serum level of interleukins in alopecia areata patients on the basis of severity (n=43) Data were expressed as mean ± SD (standard deviation), median, and range. The Kruskal-Wallis test was performed among groups. IL-17A: interleukin 17A; IL-23: interleukin 23. * The test is significant, n: number.

Interleukins	Parameter	Severity of Alopecia Areata					Test value/p-value
		S1, n (11)	S2, n (13)	S3, n (10)	S4, n (7)	S5, n (2)	-
IL-17A	Mean SD	21.17 ± 33.26	35.36 ± 33.45	52.04 ± 34.39	63.47 ± 50.33	5.21 ± 3.51	p=0.016*, H=12.14
	Median	10.5	17.1	54.4	95.3	5.21	
	Range	3.05–108.10	8.79–128.00	3.24–106.40	8.10–112.10	2.72–7.69	
IL-23	Mean SD	28.76 ± 58.04	31.87 ± 30.14	68.42 ± 60.92	90.84 ± 77.38	7.83 ± 9.30	p=0.118, H=7.36
	Median	9.9	19	69.1	111.1	7.8	
	Range	1.29–181.40	8.39–108.00	1.48–166.70	7.41–172.60	1.25–14.40	

Also, significant differences were found for IL-17A levels (p=0.044) among the clinical subtypes, with lower levels in AU (5.21 ± 3.51 pg/mL) compared to other subtypes, such as multiple patches (47.87 ± 43.79 pg/mL) and ophiasis (48.77 ± 44.76 pg/mL). In contrast, IL-23 levels did not significantly differ (p=0.378) across the clinical subtypes, ranging broadly from single patch (23.85 ± 27.76 pg/mL) to AT (67.03 ± 83.16 pg/mL) (Table 6).

**Table 6:** Comparison of serum interleukin levels stratified by clinical subtype of alopecia areata (n=43) Data were expressed as mean±SD, median, and range. The Kruskal-Wallis test was performed among groups. IL-17A: interleukin-17; IL-23A: interleukin-23. * The test is significant; n: number.

Variable		Clinical Subtypes of Alopecia Areata						Test value/p-value
Interleukin	Parameter	Single, n (11)	Multiple, n (21)	Ophiasis, n (4)	Sisaipho, n (2)	Alopecia totalis, n (2)	Alopecia universalis, n (3)	-
IL-17A	Mean ± SD	20.70 ± 24.46	47.87 ± 43.79	48.77 ± 44.76	53.37 ± 1.33	48.47 ± 46.69	5.21 ± 3.51	p=0.044*, H=13.21
	Median	11.1	27.67	40.6	52.6	27.2	5.21	
	Range	3.05–83.7	3.24–128.0	8.79–105.1	52.60–54.9	16.20-102.0	2.72–7.7	
IL-23	Mean ± SD	23.85 ± 27.76	60.06 ± 65.29	66.35 ± 68.22	42.30 ± 49.54	67.03 ± 83.16	7.83 ± 9.30	p=0.378, H=2.51
	Median	13.5	24.2	47.5	13.7	22	7.83	
	Range	1.29–81.3	1.30–181.4	13.50–156.9	13.70–99.5	16.10–163.0	1.25–14.4	

## Discussion

This study investigated the clinical characteristics, inflammatory biomarkers, and cytokine profiles of patients with AA, with particular emphasis on IL‑17A and IL‑23. No significant differences were observed in age or gender between AA patients and healthy controls, consistent with previous reports indicating that AA affects both sexes across a wide age range [17,18]. Atwa et al. reported similar mean ages between AA patients (22.68 ± 8.62 years) and controls (23.22 ± 8.95 years) [17], while Hatif et al. also found no significant differences in age or sex distribution [18]. Regarding clinical subtypes, multiple-patch alopecia was the most frequent presentation (48.8%), consistent with findings by Le et al., where 47.2% of patients had a single patch, and 30.6% had multiple patches [19]. Less common subtypes, including ophiasis and AU, were also observed, while diffuse AA was absent. These observations reinforce previous evidence that patchy forms predominate in AA. The duration of AA varied considerably, with 41.9% of patients experiencing hair loss for less than six months and 58.1% for more than six months. The mean disease duration was 24.8 months (range: 1-144 months), reflecting both acute and chronic presentations. This variability aligns with prior studies reporting disease durations ranging from 57.88 ± 24.38 days to 7.1 months [20,21].

Assessment of disease severity using the SALT score showed that 20.9% of patients had mild involvement (<25% scalp hair loss), 34.9% had moderate involvement (25-49%), and 23.3% and 16.3% had severe and very severe involvement, respectively. This distribution aligns with prior reports [21,22], though the proportion of severe cases varies across populations, suggesting potential demographic or environmental influences on disease expression.

Cytokine profiling revealed significantly elevated serum IL‑17A levels in AA patients (39.4 ± 38.3 pg/mL) compared to controls (13.7 ± 10.7 pg/mL, p < 0.001). These results corroborate previous studies reporting increased IL‑17A in AA, including Aljabali and Kuts [21], Ibrahim et al. [22], El‑Morsy et al. [23], Gautam et al. [20], Atwa et al. [17], and Loh et al. [24]. Elevated IL‑17A supports its established role in immune-mediated hair follicle destruction.

Similarly, serum IL‑23 levels were higher in AA patients (48.2 ± 57.0 pg/mL) than in controls (15.5 ± 15.5 pg/mL). Talal et al. reported comparable findings, with significantly elevated IL‑23 in AA patients [25]. Given IL‑23’s central role in Th17 cell differentiation, these findings underscore a potential IL‑23/IL‑17A axis in AA pathogenesis. However, some variability exists; Gautam et al. reported no significant difference in IL‑23 levels between patients and controls [20], highlighting potential population-specific differences.

A strong positive correlation was observed between serum IL‑17A and IL‑23 levels (rho=+0.762, p < 0.001), consistent with Gautam et al., who also reported a positive correlation (r=0.391, p=0.012) [20]. This association suggests that these cytokines may act synergistically in the autoimmune processes underlying AA.

When analyzed according to disease severity, IL‑17A levels increased with higher SALT scores, peaking in patients with very severe involvement (S4), aligning with observations by Atwa et al. [17]. In contrast, IL‑23 levels did not correlate significantly with disease severity, suggesting that IL‑17A may be a more sensitive biomarker for monitoring disease progression.

Evaluation of cytokines across clinical subtypes demonstrated significantly higher IL‑17A levels in the sisaipho subtype (53.37 ± 1.33 pg/mL), whereas IL‑23 levels remained consistent across subtypes. These findings indicate that IL‑17A may serve as a marker for more severe or extensive forms of AA, supporting prior observations [17].

Overall, this study reinforces the role of IL‑17A and the IL‑23/IL‑17A axis in AA pathogenesis and highlights the potential utility of IL‑17A as a biomarker for disease severity and subtype differentiation.

Limitations of the study

The sample size in the severe AA subtypes (S4-S5) was small, which may have reduced statistical power. Its cross-sectional design prevents assessment of longitudinal changes or causal relationships. Key potential confounders, including treatment status, disease duration, and metabolic or immune factors, were not fully controlled.

## Conclusions

The findings reveal statistically significant elevations in serum IL-17A and IL-23 levels among patients with AA compared to healthy individuals, indicating their potential roles in the disease's pathogenesis. IL-17A levels correlated with disease severity and exhibited variability across clinical subtypes, suggesting its involvement in both disease severity and phenotype diversity. Conversely, IL-23 levels remained consistently elevated across severity categories and exhibited variability across clinical subtypes, but this was not statistically significant.
